# The survival rate of patients with beta-thalassemia major and intermedia and its trends in recent years in Iran

**DOI:** 10.4178/epih.e2018048

**Published:** 2018-10-03

**Authors:** Alireza Ansari-Moghaddam, Hossein Ali Adineh, Iraj Zareban, Mehdi Mohammadi, Mahtab Maghsoodlu

**Affiliations:** 1 Health Promotion Research Center, Zahedan University of Medical Sciences, Zahedan, Iran; 2 Department of Epidemiology and Biostatistics, Iranshahr University of Medical Sciences, Iranshahr, Iran; 3 Department of Health Education, Health Promotion Research Center, Zahedan University of Medical Sciences, Zahedan, Iran; 4 Research Center, Iran Blood Transfusion Organization, Tehran, Iran

**Keywords:** Beta-thalassemia, Gender, Prevalence, Survival, Iran

## Abstract

**OBJECTIVES:**

Thalassemia is a common genetic disease in Iran, especially in the north and south of Iran. The present study sought to determine the survival rate of patients with thalassemia in highly endemic regions of Iran and its variation in patients born before and after 1971.

**METHODS:**

The present historical cohort study extracted data from the health records of patients with beta-thalassemia major, beta-thalassemia intermedia, and sickle beta-thalassemia who had presented to thalassemia treatment centers in the past years. The collected data were analyzed using the Kaplan-Meier test, the log-rank test, and the chi-square test.

**RESULTS:**

Of the total of 5,491 medical records (2,647 men and 2,634 women; mean age, 23.81±11.32 years), 3,936 belonged to patients with beta-thalassemia major, and 999 and 89 to patients with beta-thalassemia intermedia and sickle beta-thalassemia, respectively. In 467 cases, the type of thalassemia was not clear. The cumulative survival rate was calculated as 0.92, 0.83, 0.74, and 0.51 by ages 25, 35, 45, and 55, respectively. The hazard ratio of death was 4.22 (p<0.05) for beta-thalassemia major and 0.77 for beta-thalassemia intermedia (p=0.70). It was calculated as 1.45 for men patients and as 3.82 for single patients.

**CONCLUSIONS:**

The present study showed relatively high survival rates in patients with thalassemia. The survival of patients was unfavorable in poorer regions (Zahedan and Iranshahr). Factors including women gender, a higher level of education, being married, and living in metropolises decreased the risk of death at younger ages and improved survival.

## INTRODUCTION

Beta-thalassemia is an inherited hemoglobin disorder in the beta-globin chain that results in chronic hemolytic anemia. Two major forms of beta-thalassemia have been identified according to their clinical severity [1,2]. Thalassemia major (TM) manifests with severe anemia in the first year of life in newborns, while thalassemia intermedia (TI) involves clinically asymptomatic mild anemia [3]. In developing and underdeveloped countries, patients with TM die in childhood and adolescence due to the lack of public access to blood transfusions tested for viruses and timely iron removal from the body [4]. The underlying causes of death in patients with TM include iron overload and excess serum ferritin [5], which lead to cardiac disease, liver and endocrine disorders, infection, thrombosis, anemia, malignancy, and eventually death [6-9].

In the long term, untreated TI causes gallstones, pulmonary hypertension, thrombophilia, osteoporosis, and foot ulcers [10-12]. These symptoms are rare in patients with TM. Untreated TI causes cardiac problems in the fourth decade of life through hypoxia and increased pulmonary resistance, potentially leading to death [1,11,13-15].

Beta-thalassemia is widely prevalent across the Mediterranean region, Africa, and the Middle East (including Iran), and the prevalence of the mutation reaches 3 to 10% in some of these regions [2]. In Iran, beta-thalassemia is the most common form [16]; the disorder is most common along the coast of the Persian Gulf and the Caspian Sea, with a prevalence of more than 10% [17,18].

Although the prognosis and survival of thalassemia patients are improving across the world [19] and in Iran (based on results of some local studies), the prevalence of complications remains high in those affected [20-22], and cardiac disease remains the main cause of death in patients with high ferritin levels [23]. Variations in the prognosis of thalassemia patients may be due to the development of new treatments, such as bone marrow transplant or splenectomy in TI patients [7,10,24,25], or due to certain new causes of death, such as human immunodeficiency virus and hepatitis C [7].

Given the variations in the prognosis of the disease, obtaining adequate reliable information about common complications that affect the trend and prognosis of the disease in patients treated with conventional or modern methods in different regions of Iran is essential for planning and making decisions about genetic consultations and bone marrow transplantations in affected children [26].

The present study was conducted to determine the survival rate of patients with TM, TI, and sickle-thalassemia in highly endemic regions of Iran and to examine the effects of the main factors associated with survival in patients with TM and TI.

## MATERIALS AND METHODS

The present historical cohort study was conducted to determine the survival rate of patients with thalassemia born before 1971 and those born from 1971 to 2015 in Sistan and Baluchestan (Zahedan and Iranshahr), Hormozgan (Center for Special Diseases of Shahid Mohammadi Hospital), Tehran, and Mazandaran (Sari) Provinces. In Iran, the northern and southern provinces have highest frequency of thalassemia, and there are referral hospitals in the selected provinces that accept all patients from around the areas of the referral hospitals. Therefore, based on the prevalence of thalassemia and the existence of referral hospitals/centers, we selected 2 high-prevalence provinces from the north (Tehran and Mazandaran) and 2 provinces (Sistan and Baluchestan [Zahedan and Iranshahr] and Hormozgan) from the south of Iran. People with symptoms of anemia are screened for thalassemia at health provider centers, and patients suffering from thalassemia are referred to centers that provide medical services for thalassemia patients. The study population consisted of patients with TM, TI, and sickle beta-thalassemia that had medical records at the thalassemia units and were receiving medical services from them. Patients who had not presented to these medical centers in the 2 years preceding the study or who had incomplete or no records were excluded from the study.

Data about the form of thalassemia, the date of diagnosis, the initiation of iron removal from the body, and age were extracted from the patients’ records, along with some other required information. Trained investigators who were familiar with medical records visited the referral centers for thalassemia in each province, selected the records of all patients admitted from the inauguration of the center until 2015, and extracted the patients’ demographic details, such as age, gender, family history of thalassemia, date of diagnosis, and date of initiation of treatment. Certain outcomes, such as the date of death after the diagnosis of thalassemia, were determined by referring to the patients’ health records or the hospital archivists, or by asking their physicians. Subjects without valid and secure evidence of their death were considered to still be alive.

**Figure 1.**
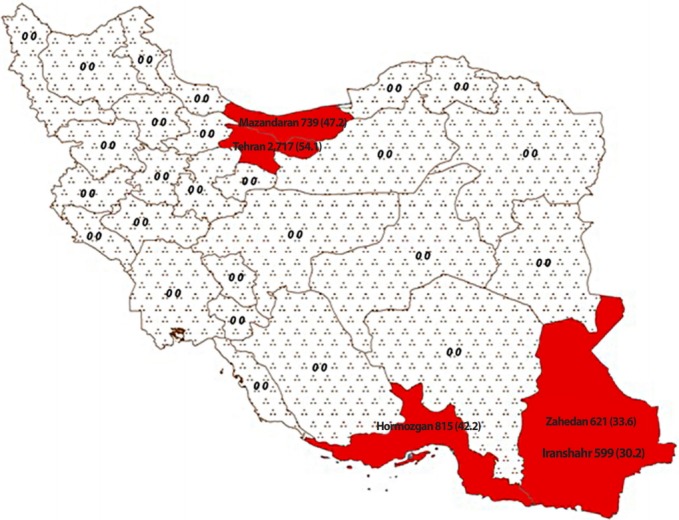
The selected provinces of Iran included in this study (name of provinces, sample size, and survival rate).

The present study examined the health records of a total of 5,491 patients from Tehran (n=2,717), Sari (n=739), Bandar-e-Abbas (Hormozgan) (n=815), Iranshahr (n=599), and Zahedan (n=621) using the consensus method (Figure 1).

Thalassemia is prevalent in the southern and northern regions of Iran. Due to the high frequency of thalassemia and the distances between cities, thalassemia patients are referred to a central hospital in each province. In the present study, all medical records from the selected provinces in the south of Iran (Hormozgan, Zahedan, and Iranshahr) and the north of Iran (Tehran and Mazandaran) were assessed to analyze the survival rate. It should be noted that Iranshahr and Zahedan are the 2 major cities in Sistan and Baluchestan Province; Iranshahr is located in the south and Zahedan in the north of the province.

### Data entry and analysis

The extracted data from the patients’ records were entered into a pre-developed checklist and then transferred to Microsoft Excel 2010 (Microsoft, Redmond, WA, USA). The survival duration of each participant was obtained by subtracting the date of birth from the date of death. The survival rate of thalassemia patients was determined using the Kaplan-Meier test; this rate was compared between the different groups using the log-rank test. Trends in patient survival over the defined period were demonstrated using Kaplan-Meier curves. We carried out a Cox regression model to determine the effect of some factors on the survival of patients; these variables were separately entered into the model. We used a significance level of 0.05 and 95% confidence intervals (CIs). The relationship between the categorical variables was assessed using the chi-square test. All the analyses were performed in SPSS version 22.0 (IBM Corp., Armonk, NY, USA).

### Ethics statements

The present study was examined and approved by the ethics committee of Zahedan University of Medical Sciences. All procedures performed in studies involving human participants were in accordance with the ethical standards of the institutional and/or national research committee and with the 1964 Helsinki declaration and its later amendments or comparable ethical standards.

## RESULTS

The present study examined 5,491 health records (2,647 [48.2%] from men and 2,634 [48.0%] from women, and 210 (3.8%) cases did not have available medical records). The mean age of the thalassemia patients was estimated as 23.81±11.32 years; it was very similar in Tehran and Sari (28.53 vs. 28.12 years), but was lower in Bandar-e-Abbas (Hormozgan) and Zahedan (18.92 and 15.88 years). Iranshahr had the youngest population of thalassemia patients, with a mean age of 11.39, which was lower than in the other cities.

The plurality of medical records (n=2,717, 49.5%) were obtained from Tehran, the capital of Iran. Additionally, 739 (13.5%) records were obtained from Sari, 815 (14.8%) from Bandar-e-Abbas (Hormozgan), 621 (11.3%) from Zahedan, and 599 (10.9%) from Iranshahr. By type of thalassemia, 3,936 (71.7%) belonged to patients with TM, 999 (2.2%) to patients with TI, and 89 (1.6%) to patients with sickle beta-thalassemia. The form of thalassemia could not be determined in 467 (8.5%) cases due to illegible, outdated, or incomplete records. In terms of the level of education, more than 60% of the TM patients, 50% of those with TI, and 70% of those with sickle beta-thalassemia had less than a high school education. Overall, 1,091 (22.5%) patients were illiterate, 1,947 (40.0%) had less than a high school education, 995 (20.5%) had a high school diploma, and 826 (17.0%) had above a high school education. Iranshahr, among the poorest cities in Iran, had the highest percentage of illiterate patients (54.5%), followed by Zahedan (19.6%) and Tehran (14.8%). Sari had the lowest number of illiterate patients (0.1%), followed by Bandar-e-Abbas (Hormozgan) (0.1%). Overall, 79.4% of the thalassemia patients were single and 20.2% were married. According to the health records, 85.2% of the TM patients, 66.0% of the TI patients, and 73.1% of the sickle beta-thalassemia patients were single.

Our findings showed that 1,666 (30.3%) patients were blood relatives of their spouse, while 1,519 (27.7%) had no such relationship to their spouse. The ratio of patients who were related by blood to their spouse was similar for all the different forms of thalassemia; however, this ratio differed by city. In Zahedan, most thalassemia patients (72.1 vs. 27.9%) reported a blood relationship to their spouse; in Bandar-e-Abbas (Hormozgan), this ratio (56.7%) was lower than in Zahedan, but higher than in all the other cities examined; in Sari, it was 32.5% (vs. the 67.5% who were not related by blood to their spouse); in Tehran, it was 50.7% (vs. 49.2%) (Table 1).

### Survival of thalassemia patients

Table 2 shows the survival of patients in 5-year intervals. The survival rate varied with the number of deaths in each age group, with the highest number of deaths occurring around age 45. The survival rate was calculated to be 0.92 in the 45-50 age group and as 0.75 in the 50-55 age group. The cumulative survival rate from birth until 10 years old was 99%. After reaching the age of 20 years, 88% of the patients survived until 30 years, 74% survived until 45, 68% survived until 50, and 51% survived until 55 years old. The age of 242 patients was inadvertently not extracted or had not been recorded.

### The survival curve of patients born before and after 1971

**Figure 2.**
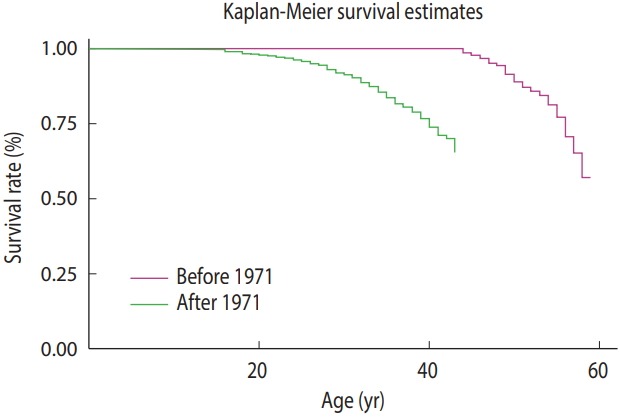
The survival curve of patients born before and after 1971.

Figure 2 presents the survival rate until various ages for patients born before and after 1971. As shown, 100% of the patients born before 1971 survived until age 35, and then their survival declined and dropped to 93% by age 45 and to 73% by age 55. In contrast, 100% of the patients born after 1971 survived until age 10, and then their survival dropped to 98% by age 15 and to 85% by age 35.

### Estimated survival by form of thalassemia, place of residence, and gender

The mean estimated survival of thalassemia patients in Iran (Tehran, Mazandaran, Bandar-e-Abbas [Hormozgan], Zahedan and Iranshahr) was calculated as 52.42 years using the Kaplan-Meier test (95% CI, 51.61 to 53.23).

**Figure 3.**
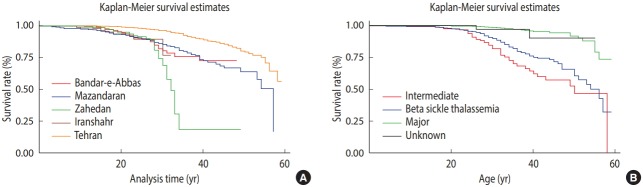
Kaplan-Meier survival estimates by (A) city and (B) different forms of thalassemia.

The rate of survival differed significantly between the patients with TM, TI, sickle, and unidentified thalassemia (p<0.001). The patients with TI tended to survive the longest (55.59 years; 95% CI, 55.65 to 57.52), followed by the patients with sickle beta-thalassemia (53.00 years; 95% CI, 50.54 to 55.66), while the patients with TM died earlier (50.07 years; 95% CI, 48.54 to 51.60). The form of thalassemia was not recorded in the checklist for a number of patients, whose mean age at death was calculated as 46.26 years, ranging from 43.08 to 49.44 years. Figure 3 and Table 3 show trends in survival by city and by form of thalassemia, respectively. The proportional hazard assumption was assessed by chi-square test and graphical plots, since the slight heterogeneity of variance that we observed in the relevant categories led to a slight violation of this assumption (p=0.06 and 0.07 for Figure 3).

Survival differed significantly across cities (p<0.001), with the patients from Tehran surviving longest and those from Iranshahr having the shortest survival (54.10 vs. 30.23 years). Following Tehran, the patients from Mazandaran survived longest (47.28 years). As shown in Table 3 and Figure 3, the patients from Zahedan (33.59 years) and Iranshahr (30.23 years) had the shortest survival. The log-rank test showed a significant difference in the mean survival rate between men and women (p=0.001). The mean survival was 53.49 years in women, which was lower than that in men (51.47 years).

### Median survival by form of thalassemia and place of residence

The median survival was 58 years in the samples studied. This value differed across cities; it was 32 years in Zahedan, 54 in Mazandaran, 45 in Bandar-e-Abbas (Hormozgan) and 55 in Tehran. The median survival was 57 years in patients with TM, 55 in those with TI or sickle beta-thalassemia, and 49 in those with unidentified thalassemia (Table 3).

### The effects of gender, city of residence, and form of thalassemia on the survival of thalassemia patients

The Cox regression test was used to examine the effects of gender, city of residence, and form of thalassemia on survival. The unadjusted hazard ratio (HR) was calculated for each variable. As shown in Table 4, Tehran was chosen as the reference group, and the HR for death was calculated for the remaining cities. The risk of death was higher in Zahedan and Iranshahr (6.25 and 5.42) than in Bandar-e-Abbas (Hormozgan) and Sari. The HR for death was also calculated for men and women; this ratio was higher in men than in women by 45% (p=0.001). Moreover, patients with TM had a higher risk of death than those with sickle beta-thalassemia (HR, 4.22), and the risk was lower in those with TI than in those with sickle beta-thalassemia (HR, 0.77). A low level of education also significantly affected the risk of death, as the HR for death was threefold higher in illiterate patients than in those with above a high school education (HR, 2.49; p=0.03). The corresponding HRs were 3.80 for patients with below a high school education and 1.80 for those with a high school diploma. Examining the unadjusted effect of marital status on the HR for death showed that single patients had a threefold higher risk of death than married patients (p<0.001). The number of widowed patients was very low in this study (n=4), and they were therefore excluded from the analysis; the HR for death was likewise not significant in divorced patients due to their low number. Despite the role of the discussed variables in increasing the HR for death, age at diagnosis, and date of initiation of iron removal from the body had an inverse relationship; in other words, they had a protective relationship with survival. The HR was calculated as 0.99 for the date of diagnosis and 1.00 for the initiation of iron removal from the body (p<0.001).

## DISCUSSION

The present study examined 5,491 records of thalassemia patients. The overall mean and median survival of the patients were calculated as 52.42 and 58.00 years, respectively, which seem to be higher than the mean survival of patients reported in a study conducted only in Tehran (41.97 years) [27]. The higher overall survival in the present study may have been because of differences in survival across cities and according to the type of thalassemia. A significant difference was observed in the mean survival between men (51.47 years) and women (53.49 years), suggesting a longer survival in women, which is consistent with the results obtained in other studies; however, the mean survival reported for men and women in the present study is longer than has been reported in other studies [28]. The differences in the survival rates reported in various studies may be attributed to the smaller sample size used in the studies and calculating survival rate only in beta-thalassemia or intermedia thalassemia patients. The present study showed that 99% of the patients lived until 10, 97% until 15, 95% until 20, and 92% until 25 years old. The survival rates reported for these age intervals are higher than those reported in studies conducted in other parts of Iran (Shiraz, Hamadan, and Sistan and Baluchestan) [28-30]. In studies conducted by Zamani et al. [28] and Rajaeefard et al. [29], 88 and 85% of the patients lived until age 20, compared to the 95% rate reported in the present study. The improved patient survival in the present study may be explained by timely iron removal from the body and better patient care [31,32]. The survival rate to the age of 30 was estimated as roughly 41% in Ahvaz (a province in southwest Iran), which is lower than our estimation (88%), but this discrepancy may be explained by differences in patients’ place of residence [33]. Studies conducted in other countries over the last few years reported that the survival rate for thalassemia patients was 89% until the age of 20 and 82% until the age of 25 [26], which is similar to the results obtained in studies previously conducted in Iran; however, the rates may have gradually improved in recent years. Nonetheless, as shown in the survival curves, the patients born after 1971 showed a lower survival at older ages than those born before 1971; for instance, the survival rate was 99% until 40 years in patients born before 1971, compared to 85% in patients born after 1971. The reason for the lower survival of patients born in recent years is that we estimated the overall survival rate without adjusting for the place of residence. Since inhabitants of the south of Iran (Iranshahr and Zahedan) do not have enough access to health care and medical surveillance, their low survival rate affected the overall estimation of survival. In addition, our findings may have been affected by selection bias, in which patients with severe forms of disease may have died before being diagnosed and having medical records made; therefore, patients with better survival remained in the cohort born before 1971, whereas improvements in diagnostic tools might have led to diagnosis of the severe forms of thalassemia in patients born after 1971, resulting in a lower estimated survival. Furthermore, the level of access to health services and health literacy of patients may have contributed to differences in the survival rate. Before 1971, the disease registry in Iran was not as complete as after 1971. In that period of time, there was limited access to health services. Therefore, selected patients with good knowledge and perceptions of the disease, as well as relatively high socioeconomic conditions, may have been more likely to seek care in the health system.

The mean survival was 50.07 years for patients with TM and 56.59 and 53.10 years for patients with TI and sickle beta-thalassemia, respectively. TM patients appear to be at a greater risk of complications and death due to the severity of the disease and their higher need for transfusion and the subsequent iron overload in their body [11]. The mean survival of patients differed across cities, and the highest survival was observed in Tehran and the lowest in Iranshahr (54.10 vs. 30.23 years). It appears that patients residing in Tehran were in a better condition due to the early diagnosis of their disease, the earlier iron removal from their body, and the better care they received in the course of illness, while only 7 out of 590 patients and 27 out of 530 patients remained alive until 30 years old in Iranshahr and Zahedan, respectively. This suggests that patients in these cities receive later diagnoses and poorer care.

The survival of thalassemia patients is affected by various factors, including gender, which was examined in the present study. Although some studies have shown that the survival of patients was not affected by gender [33], others have confirmed the effect of gender on patient survival [26]. According to the present findings, the chances of death were 45% higher in men than in women, which is consistent with the results obtained in a study conducted in Hamadan [28]. The survival calculated in the life table and the Cox regression test showed that, in addition to men gender, having TM was associated with a higher risk of death compared to all other forms of the disease (HR, 4.22 for TM and 0.77 for TI). Patients with TM are at a higher risk of death due to their more critical condition, greater need for transfusion and the subsequent iron overload in their body, and various other complications. Another factor that indirectly affects the survival of thalassemia patients is their place of residence. In the present study, the risk of death in thalassemia patients was 5 and 6 times higher in Iranshahr and Zahedan than in Tehran (p<0.001). The risk of death was also 3.30 and 4.00 times higher in Sari and Bandar-e-Abbas (Hormozgan) than in Tehran (p<0.001). The differences in the survival rate across cities may have been due to differences in the available health care services, timeliness of diagnoses, regular and accurate treatment measures, and regular monitoring and follow-up by medical teams.

In the present study, age at diagnosis was found to be inversely related to the risk of death. In other words, the earlier the disease is

diagnosed, the longer patients will survive [20]. The improved survival of patients may be caused by early care procedures. Another protective factor with an inverse relationship with the risk of death was found to be the initiation of deferoxamine therapy. The early initiation of deferoxamine therapy reduces damage to the body organs and thereby prolongs survival [34].

The substantial sample size of 5,491, the nationwide coverage, and the investigation of all the existing records in the examined cities are strengths of this study. The main limitations were incomplete records (especially in Iranshahr), which caused missing data, difficulty accessing the records of deceased patients, and the geographical dispersion of the selected areas of study. Another point that should be considered in the interpretation and application of our results is that some patients with health records in Tehran appeared to have been residents of other cities, such as Sari and Bandar-e-Abbas (Hormozgan), which may have caused repeated entry and analysis of certain individuals’ details. Furthermore, regarding the study of medical records from hospitals, the possibility of selection bias must be considered when interpreting the results. According to the results obtained, the survival rate was 50% by age 55. The overall mean survival of patients was 52.42 years, and the mean survival of patients with TM was 50.07 years. Patients living in Tehran survived longer than those in other cities, and the patients Iranshahr and Zahedan had an unfavorable lifespan. Gender was found to affect the risk of death, with men having a 45% higher risk of death than women. The risk of death was also 4.22 times higher in patients with TM than in those with the sickle form of the disease. A higher level of education, women gender, being married as opposed to being single, and living in metropolises (due to the better healthcare services) increased patient survival.

## Figures and Tables

**Table 1.** The patients’ descriptive data by form of thalassemia

Variables	Type of beta-thalassemia				Total
	Major	Intermedia	Sickle	Unknown cases^1^	
Age (yr)	21.56±10.28	30.72±11.52	25.30±11.44	24.88±12.58	23.50±11.24
Gender^2^					
Men	1,957 (51.1)	445 (47.0)	39 (44.3)	206 (49.8)	2,647 (50.1)
Women	1,876 (48.9)	501 (53.0)	49 (55.7)	208 (50.2)	2,634 (49.9)
Residence					
Tehran	1,695 (43.1)	694 (69.5)	40 (44.9)	144 (61.7)	2,717 (49.5)
Sari	470 (11.9)	125 (12.5)	-	144 (30.8)	739 (13.5)
Iranshahr	573 (14.6)	-	-	26 (5.6)	599 (10.9)
Zahedan	613 (15.6)	4 (0.4)	-	4 (0.9)	621 (11.3)
Hormozgan	585 (14.9)	176 (17.6)	49 (55.1)	5 (1.0)	815 (14.8)
Education^3^					
Illiterate	971 (27.0)	60 (6.7)	8 (9.5)	52 (18.4)	1,091 (40.0)
<Diploma	1,430 (39.7)	367 (41.2)	51 (60.7)	99 (35.0)	1,947 (20.5)
Diploma^4^	652 (18.1)	256 (28.7)	18 (21.4)	69 (24.4)	995 (20.5)
>Diploma	548 (15.2)	208 (23.3)	7 (8.3)	63 (22.3)	826 (17.0)
Marriage^5^					
Married	427 (14.4)	304 (33.6)	21 (26.9)	108 (36.7)	860 (20.2)
Single	2,534 (85.2)	597 (66.0)	57 (73.1)	186 (63.3)	3,374 (79.4)
Divorced	12 (0.4)	4 (0.4)	-	-	16 (0.4)
Consanguinity^6^					
Yes	1,299 (53.7)	284 (47.1)	38 (57.6)	45 (45.5)	1,666 (30.3)
No	1,119 (46.3)	319 (52.9)	28 (42.4)	53 (54.1)	1,519 (27.7)
Missing					2,306 (42.0)

Values are presented as number (%) or mean±standard deviation.

1 Refers to cases where we could not find the type of thalassemia in medical records.

2 210 patients were missing data for gender.

3 632 patients did not have information about their education level.

4 Subjects who had formal education for 12 years in Iran.

5 1,241 patients were missing data.

6 Patients whose parents were blood relations, apart from the marriage connection.

**Table 2.** The life table of thalassemia patients

Age (yr)	Living patients (n)^1^	Proportion of survival at each interval	Cumulative proportion of survival at end of the interval
0	5,249	1.00	1.00
5	5,018	1.00	1.00
10	4,573	1.00	0.99
15	4,080	0.98	0.97
20	3,426	0.98	0.95
25	2,632	0.97	0.92
30	1,549	0.95	0.88
35	813	0.95	0.83
40	395	0.95	0.79
45	200	0.93	0.74
50	104	0.92	0.68
55	35	0.75	0.51

1 Number of people who were alive at each defined time period.

**Table 3.** Mean and median survival by demographic factors

Variables	Mean survival			Median survival			
	Survival estimate	95% CI		Survival estimate	95% CI		p-value
		LL	UL		LL	UL	
Type of thalassemia							<0.001
Major	50.07	48.54	51.60	57.00	51.32	62.67	
Intermediate	56.59	55.65	57.52	55.00	NA		
Sickle	53.10	50.54	55.66	55.00	NA		
Not defined	46.26	43.08	49.44	49 .00	NA		
Residence (city)							<0.001
Tehran	54.10	53.22	54.97	55.00	NA		
Mazandaran	47.28	45.61	48.95	54.00	51.62	56.37	
Bandar-e-Abbas	42.21	40.82	43.60	45.00	NA		
Zahedan	33.59	30.55	36.63	32.00	30.74	33.25	
Iranshahr	30.23	29.34	31.11	30.00	NA		
Gender							0.001
Men	51.47	50.33	52.01		NA		
Women	53.49	52.39	54.58		NA		
Total	52.42	51.61	53.23	58.00	NA		-

CI, confidence interval; LL, lower limit; UL, upper limit; NA, not applicable (since censored data).

**Table 4.** Summary associations with incident death among thalassemia patients

Variables	HR (95% CI)	p-value	Adjusted HR (95% CI)^1^	p-value
Age of diagnosis	0.99 (0.98, 0.99)	<0.001	1.00 (1.00, 1.00)	0.89
Age of starting chelation	1.00 (0.99, 1.00)	<0.001	1.00 (1.00, 1.00)	0.93
Type of thalassemia				
Sickle beta thalassemia	1.00 (reference)		1.00 (reference)	
Major	4.22 (1.05, 17.01)	0.04	2.40 (0.14, 40.00)	0.53
Intermediate	0.77 (0.18, 3.27)	0.70	1.20 (0.15, 9.60)	0.84
Not defined	7.90 (1.92, 33.00)	0.004	3.80 (0.52, 28.40)	0.18
Residence				
Tehran	1.00 (reference)		1.00 (reference)	-
Sari (Mazandaran)	3.30 (2.59, 4.25)	<0.001	0.60 (0.20, 2.10)	0.48
Hormozgan	4.00 (2.99, 5.48)		5.30 (3.10, 9.05)	<0.001
Iranshahr	5.40 (3.20, 9.18)		5.70 (2.60, 12.00)	<0.001
Zahedan	6.20 (4.33, 9.03)		5.20 (2.80, 9.60)	<0.001
Gender				
Women	1.00 (reference)		1.00 (reference)	
Men	1.40 (1.17, 1.81)	0.001	1.20 (0.82, 1.70)	0.31
Education level				
Illiterate	2.49 (1.09, 5.66)	0.03	0.80 (0.23, 3.20)	0.84
< Diploma	3.80 (2.36, 6.19)	<0.001	2.30 (1.20, 4.50)	0.01
Diploma^2^	1.80 (1.09, 3.14)	0.02	1.30 (0.66, 2.68)	0.41
>Diploma	1.00 (reference)		1.00 (reference)	-
Marriage				
Married	1.00 (reference)		1.00 (reference)	
Single	3.80 (2.52, 5.79)	<0.001	2.80 (1.40, 5.60)	0.002
Divorced	NA	0.90	NA	-

HR, hazard ratio; CI, confidence interval; NA, not applicable (since the corresponding category did not include enough subjects).

1 The multivariate analysis included all variables listed in Table 3. The enter method was selected for estimating the HR.

2 Subjects who had 12 years of formal education in Iran.
